# Metanephric adenofibroma of the left kidney in an adult patient

**DOI:** 10.1016/j.eucr.2026.103385

**Published:** 2026-02-19

**Authors:** Austin Erickson, Audra Garrigan, Trushar Patel

**Affiliations:** a University of South Florida Health Morsani College of Medicine, Tampa, FL, USA; b Department of Urology, University of South Florida, Tampa, FL, USA

**Keywords:** Metanephric adenofibroma, Metanephric tumor, Renal, Robotic partial nephrectomy

## Abstract

Metanephric adenofibroma is a rare, benign renal neoplasm arising from the embryonic metanephric tissue, which would typically give rise to normal kidney parenchyma. Despite classical presentation in pediatric patients, we present a case of a 29-year-old female with 6 months of intermittent left upper quadrant pain found to have a 3.8cm left renal mass, partially extending into the collecting system of the superior pole. The patient underwent robotic partial nephrectomy, and histopathology confirmed a diagnosis of metanephric adenofibroma with negative surgical margins. The patient had an uneventful recovery, and requires no acute follow-up, given the benign nature of her diagnosis.

## 1 Introduction

Metanephric adenofibroma (MAF) is a rare, benign, and biphasic renal neoplasm that presents grossly identical to other pediatric renal masses, most significantly Wilms tumor. The tumor is composed of a bland spindle cell stromal component and an epithelial component, and this distinctive biphasic architecture places it along a spectrum of metanephric tumors, between metanephric adenoma and metanephric stromal tumor.^1^^,^^2^ The tumor can be identified across a wide age range, though adult cases are rare; the mean age at presentation is approximately 13 years, with a female predominance of roughly 2:1.^1^^,^^3^ The clinical presentation varies, and may include abdominal mass, hematuria, polycythemia, or hypertension, although incidental detection on imaging may also occur.^1^^,^^3^

MAF represents a rare subgroup of renal masses, with a limited number of cases described in the literature. Despite being more common among pediatric populations, a comprehensive review identified only 20 cases of MAF.^4^ Despite the benign character of these tumors, MAF is frequently misdiagnosed as Wilms tumor, which often leads to unnecessary chemotherapy in many patients.^2^^,^^5^^,^^6^ This is likely due to the inability of imaging studies, such as magnetic resonance imaging (MRI), to distinguish between the two. No tumor recurrence has ever been reported in cases of pure MAF, confirming its benign nature.^2^^,^^3^

Immunohistochemical staining plays a key role in distinguishing MAF from other possible differential diagnoses. The epithelial component of the tumor, more closely resembling metanephric adenoma (MA), stains strongly for WT1 and CD57 and negatively for CK7 and AMACR, which is used to separate it from a renal cell carcinoma or an epithelial predominant nephroblastoma.^7^^,^^8^ The component more closely resembling metanephric stromal tumor (MST) may stain variably with CD34.^6^ Notably, the BRAF V600E mutation has emerged as a significant molecular marker of metanephric adenomas and adenofibromas, with one series of pediatric MAFs harboring the mutation in 100% of tumors reviewed.^9^^,^^10^ Given the rarity of BRAF V600E mutations in common renal tumors, such as renal cell carcinoma or Wilms tumor, immunohistochemistry for this specific mutation has become a valuable diagnostic tool.^9^^,^^10^

We describe an uncommon case of a 29-year-old female with MAF isolated to the upper pole of the left kidney in close proximity to the collecting system. The patient underwent left robotic partial nephrectomy and achieved a margin-negative resection. This case highlights the diagnostic evaluation and management of small renal masses in young individuals.

## 2 Case presentation

**Fig. 1:**
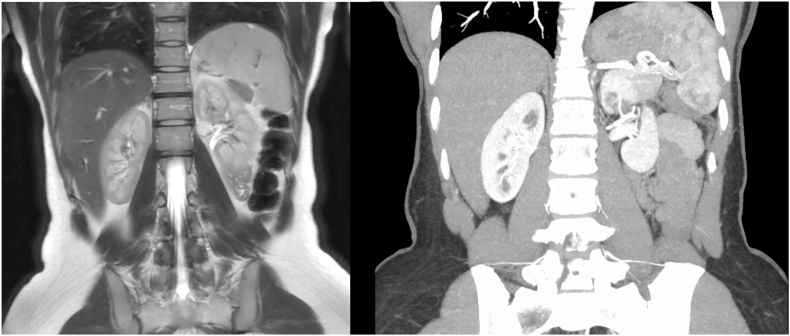
Preoperative imaging studies including MRI showing the mass found in the left kidney, which was measured to be 3.7cm (Left), and CT angiogram of accessory branching arteries to the tumor (Right).

A 29-year-old female no significant past medical history presented to the emergency department with a 6-month history of waxing and waning left upper quadrant pain and sudden onset dizziness. Laboratory testing and physical exam were unremarkable. Preoperative contrast-enhanced computer tomography (CT) scan of the abdomen and pelvis revealed a ∼3cm heterogeneously enhancing, partially exophytic lesion in the superior left renal pole. This was better characterized using preoperative MRI, which estimated the lesion to be 3.7cm and concerning for malignancy (Fig. 1). The patient had no significant past medical or surgical history and denied a family history of renal cancers or syndromes.

After lengthy discussion of treatment options, the patient opted to undergo robotic partial nephrectomy. The size of the mass required complete mobilization of the posterior upper to mid pole to visualize the extent of the mass, which was determined under ultrasound guidance. Partial nephrectomy required ligation of accessory branching arteries that fed into the tumor directly off the left renal artery (Fig. 1). Once this was done, the partial nephrectomy was done in a standard fashion using a two layered closure. After the partial nephrectomy was completed, a lateral nephropexy was performed. Estimated blood loss was 50mL, and the ischemia time was 24 min. The postoperative course was unremarkable, and the patient was discharged home on post op day 1.

**Fig. 2:**
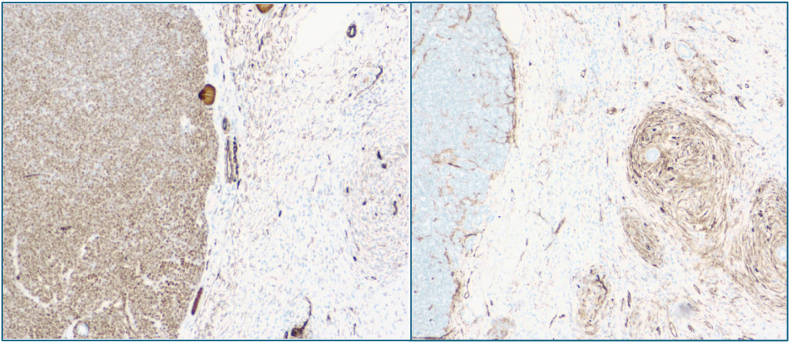
WT1 positive in the epithelial component and negative in spindle component (Left); CD34 positive in the spindle component and negative in the epithelial component (Right).

Gross pathology showed a tan-white, somewhat lobulated mass measuring 3.8cm at its largest dimension. The mass was non-invasive into neighboring adipose tissue and was contained within a bulging capsule. Renal margins were found to be unremarkable and negative for any neoplastic changes. Initial histologic examination displayed biphasic components, containing both spindle and epithelial components. Immunohistochemical staining was WT1 positive in the epithelial component and CD34 positive in the spindle component (Fig. 2). Additionally, there was positive staining for the BRAFV600E mutation, which is a notably distinguishing stain for MAF. Finally, negative staining throughout for AMACR and CK7 rules out the possibility of renal cell carcinoma or Wilms tumor. A final diagnosis of metanephric adenofibroma confined to the kidney was made. The patient was informed of the benign course of this mass and was found to be doing well two weeks after surgery.

## 3 Discussion

MAF is a rare and benign renal neoplasm within the spectrum of metanephric tumors, characterized by a distinctive biphasic combination of stromal and epithelial elements. While the majority of reported cases occur in pediatric populations, there are rare cases of adult presentation, which contributes to the diagnostic uncertainty of solitary renal masses on radiologic imaging modalities. This case adds to limited adult literature while underscoring the value of histopathologic and immunohistochemical evaluation in arriving at the correct diagnosis.

Clinically, MAF presents a complex diagnostic challenge, as imaging findings are incredibly nonspecific and virtually indistinguishable from renal cell carcinoma or Wilms tumor, another common pediatric tumor. Given the limitation of imaging studies in this case, another diagnostic possibility might have been percutaneous biopsy. However, given the biphasic nature of MAF, percutaneous biopsy may be limited by sampling error in this case, as epithelial and stromal components may be unevenly distributed throughout the tumor.^5^ Particularly, when it is considered that percutaneous biopsy shows 75% diagnostic accuracy in the homogenous relative of MAF, metanephric adenoma.^11^ In this case, a heterogeneously enhancing mass was seen, concerning for malignancy, prompting surgical intervention as both a diagnostic and treatment modality.

Histopathology remains the cornerstone of diagnosis in these tumors. The defining feature of MAF is the presence of both epithelial and bland, non-mitotic spindle cell stromal components without necrosis.^1^^,^^2^ The diagnosis was refined using immunohistochemistry, given that the absence of AMACR and CK7 virtually dismiss the possibility of papillary renal cell carcinoma, nephroblastoma, or Wilms tumor. Furthermore, presence of mixed WT1 positive epithelial component and CD34 positive stromal component are essentially diagnostic of MAF. This is additionally reinforced by the identification of BRAF V600E mutations within the tumor, which is a highly specific marker for MAF.^9^^,^^10^ All of these key identifying features on histopathology advised sound clinical management, as they supported the benign classification of this tumor.

This case is notable for several reasons, including the adult presentation of an already rare tumor, successful minimally invasive and kidney sparing management, and the diagnostic specificity provided by histopathology. This case further reinforces the need for awareness by clinicians of metanephric tumors, MAF in particular, to avoid unnecessary adjuvant chemotherapy or overly aggressive surgical treatment without adequate diagnostic certainty. Additionally, it adds to the documentation of these rare cases in the literature so that they may be further considered in the evaluation of renal masses in young adults, not just pediatric patients.

## 4 Conclusion

Metanephric adenofibroma is an uncommon subgroup among the already exceedingly rare metanephric tumors. Despite classical presentation among pediatric patients, we presented a case in a 29-year-old, otherwise healthy, female patient. The combination of such an unusual tumor found beyond its traditional demographics represents one in only a handful of cases documented in medical literature.

## CRediT authorship contribution statement

**Austin Erickson:** Data curation, Writing – original draft. **Audra Garrigan:** Data curation. **Trushar Patel:** Conceptualization, Data curation, Supervision, Writing – review & editing.

## Ethics statement

This study was deemed exempt from Institutional Review Board review.

## Funding/support

None.

## Conflict of interest disclosures

The Authors have no conflicts of interest to disclose.
